# Self-Assembly and Applications of Amphiphilic Hybrid POSS Copolymers

**DOI:** 10.3390/molecules23102481

**Published:** 2018-09-27

**Authors:** Hong Chi, Mingyue Wang, Yiting Xiao, Fuke Wang, Joshy K.S

**Affiliations:** 1 Shandong Provincial Key Laboratory of Molecular Engineering, School of Chemistry of Pharmaceutical Engineering, Qilu University of Technology (Shandong Academy of Sciences), Jinan 250353, China; Silvia9607@163.com (M.W.); Olivia9802@163.com (Y.X.); 2 Polymeric Materials Department, Institute of Materials Research and Engineering, Agency for Science, Technology and Research (A*STAR), 2 Fusionopolis Way, #08-03 Innovis, Singapore 138634, Singapore; wangf@imre.a-star.edu.sg; 3 International and Inter University Centre for Nanoscience and Nanotechnology, Mahatma Gandhi University, Kottayam 686 560, Kerala, India; joshyk.s@gmail.com

**Keywords:** self-assembly, POSS, copolymer, amphiphile

## Abstract

Understanding the mechanism of molecular self-assembly to form well-organized nanostructures is essential in the field of supramolecular chemistry. Particularly, amphiphilic copolymers incorporated with polyhedral oligomeric silsesquioxanes (POSSs) have been one of the most promising materials in material science, engineering, and biomedical fields. In this review, new ideas and research works which have been carried out over the last several years in this relatively new area with a main focus on their mechanism in self-assembly and applications are discussed. In addition, insights into the unique role of POSSs in synthesis, microphase separation, and confined size were encompassed. Finally, perspectives and challenges related to the further advancement of POSS-based amphiphilics are discussed, followed by the proposed design considerations to address the challenges that we may face in the future.

## 1. Introduction

Recent advances in amphiphilic copolymers have created a new surge of interest in the development of nanoscience, because many intelligent functions are directly determined by their shapes and dimensions [1,2,3]. Self-assembly of an amphiphilic copolymer from a single molecule to functionally architectured copolymers is an efficient strategy to create competent products for applications in drug delivery [4,5,6,7,8], sensors [9,10], bioimaging [11,12,13], nanoreactors [14,15,16], cosmetics [17,18,19], and dispersant technology [20,21,22]. Among these new amphiphiles, the incorporation of polyhedral oligomeric silsesquioxanes (POSSs) into amphiphilic polymers to obtain improved performances has been attracting particular attention because of the unique and interesting hybrid structures of POSSs.

POSSs represent the smallest hybrid silica with the formula of (RSiO_1.5_)_n_ (n = 6, 8, 12, etc.) and diameter ranging from 1 to 3 nm [23]. The size of POSSs depends on the surrounding R groups, where R could be a hydrogen atom or organic functional groups which could be precisely functionalized via the living/controlled polymerization techniques [24]. POSSs have been reported to construct hybrid polymers with well-defined structures, including telechelic-shaped [25,26,27] and star-shaped [28,29,30] polymers, dendrimers [31,32,33], block copolymers [34,35,36], and alternative copolymers [37,38]. These interesting structures and properties of POSSs make them widely used in hybrid materials [39,40], drug delivery [41,42], biomedical applications [43,44], catalytic supports [45,46], and so on [47,48,49], which have been reviewed extensively [47,50,51,52]. The focus of the present review will be on how to build amphiphilic block copolymers using POSSs to modulate the self-assembly of polymer chains and improve performances.

The superhydrophobic POSSs could provide the strong aggregation tendency useful for the controllable assembly and confined motion of polymer chains to the required nanometer size and tailored properties [53,54,55,56]. Self-assembly of such amphiphiles is normally driven by a combination of attractive and repulsive forces between the POSS segments and the organic segments. Zhang and coworkers synthesized pentatelechelic POSSs carrying poly(acrylic acid) (Glu–PAA–POSS_5_) through atom transfer radical polymerization and a “click” reaction [57]. The Glu–PAA–POSS_5_ self-assembled into giant capsules in water due to the hydrophobic POSS ending groups having the tendency to aggregate in aqueous solution, especially in the dilute solution. Several macromolecules of Glu–PAA–POSS_5_ could even form big assembled aggregates between intramolecular POSS units. 

The biocompatibility and nontoxicity of POSSs has attracted extensive research on their potential applications such as cytocompatibility [58,59], construction of capillary beds [60], and antithrombogenicity [61,62], etc. Ata and coworkers incorporated methacryloisobutyl POSS (MAPOSS) into hydrogel to improve hemocompatibility and lower platelet adhesion on the hydrogel surface. Results indicated that the presence of POSS could help to reduce the platelet adhesion and improve cytocompatibility and red blood cell compatibility because of the hydrophobicity and the mushroom-like surface topography [62]. 

Moreover, due to the special composition and cage-like nanostructure of POSSs, not only do they have many necessary properties such as oxidation resistance and thermal stability [63], but they could also strengthen the mechanical properties [64], enhance the stability of micelles, and influence viscoelastic and homogeneous properties of materials [65,66,67,68,69,70]. As a typical example, Fan and coworkers reported a dendritic structure with a POSS as the core and branched copolymers of poly(acrylic acid) (PAA), poly(l-lactide) (PLLA), and poly(ethylene glycol) (PEG) as the shell. Interestingly, this (PAA–(PLLA–PEG)_4_)_8_ could form unimolecular micelles and nanorods with great structural stability due to the existence of the POSS [71]. Furthermore, forming stable structure in aqueous solution, a well-defined amphiphilic dendritic copolymer POSS–(G_3_–PLLA-*b*-PEO–COOH)_8_ was self-assembled by single-molecule micelles with a hydrophilic poly(ethylene oxide) (PEO) shell and hydrophobic dendritic PLLA core [72]. To stabilize quantum dots (QDs) in water, Rizvi and coworkers prepared poly(carbonate–urea) urethane (POSS–PCU) amphiphilic block copolymer micelles to encapsulate QDs. The coated QDs showed both colloidal stability and high photostability, making them a promising candidate for long-term imaging applications with prolonged photostability requirements [73].

Herein, with the aim to illustrate how to take advantage of the specific structures and physical properties of POSSs in building functional materials, we summarize recent literature on the POSS-based amphiphilic copolymers on the mechanism of self-assembly and some applications, particularly in the area of drug delivery, photodynamic therapy, coatings, Langmuir Blodgett (LB) films, and sensing. 

## 2. Mechanisms of Self-Assembly of POSS-Based Amphiphilic Copolymers

Self-assembly is a process by which components form ordered arrangements or structures spontaneously [74]. The structures vary depending on the type of substance used and the environment in which they are located [75]. Recently, there has been tremendous interest in hybridized amphiphilic polymers with POSS nanocages [76,77,78,79]. Thus, a variety of interesting studies have been conducted to develop new synthetic protocols and explore their self-assembly behavior. Different structures such as vesicles, micelles, films, and so on can be obtained by different POSS copolymers in different solutions. Fundamentally, the formation of these assemblies depends on the force balance of both hydrophilic and hydrophobic parts arising from the shapes, sizes, sequences, and relative properties. For example, the phase behavior of amphiphiles is defined by the molecular shape that can be quantitatively expressed via the critical packing parameter *p* = *v*/*al*, where *v* stands for the effective volume of the hydrophobic parts, *a* represents the effective hydrophilic part surface area, and *l* is the maximum effective length [80]. Cheng and coworkers have produced a series of famous and important works starting from 2010 on POSS amphiphilic materials and their assemblies [26,81,82,83]. Recently, they found that difference between phase-separated nanostructures could be determined by precisely controlling the sequence and the number of incorporated POSS cages (composition), because the sequence of the symmetry could affect the cross-sectional areas of the hydrophobic/hydrophilic POSS domains [84]. In another work, they also found that both the compositional variation and specific sequences could induce unconventional phase formation from specifically designed chain-like giant molecules. To study the sequence–phase relationships and the sequences’ effect, they synthesized a variety of amphiphilic giant molecules by interconnecting both hydrophobic BPOSS (~1 kDa giant molecule with iso-butyl-POSS) and hydrophilic DPOSS (~1.5 kDa giant molecule with hydroxyl-POSS) nanoparticles in precisely defined sequences. Driven by the strong collective hydrogen bonding and nanophase separation, the assemblies were constructed by a core of DPOSS nanoparticles (NPs) together and covered with a thick shell formed by BPOSS NPs. The distinct locations of DPOSS directed the giant molecules into different conformations, leading to the formation of different supramolecular lattices [85]. The self-assembly of POSS-based amphiphilic copolymers is normally obtained by dispersing copolymers in an optimal solvent and then adding to water with sonication or stirring, and the assemblies are obtained when solutions are dialyzed followed by filtration.

Meanwhile, there are many kinds of methods by which to synthesize the amphiphilic hybrid POSS copolymer, including atom transfer radical polymerization (ATRP), reversible addition fragmentation chain transfer (RAFT), ring-opening solution polymerization (ROP), click chemistry [86], and ordinary radical polymerization. Each abovementioned method has its advantages and can obtain the well-defined POSS-containing copolymer. We will introduce these methods with the resulting assemblies and mechanisms according to structural features.

### 2.1. Stimuli-Responsive Micelles

The micelle is the most common self-assembled structure of POSS copolymers, with a variety of morphologies, such as the spherical micelle, necklace-like micelle, and rod-like micelle, among which the spherical micelle is the most frequently used and can be divided into pH-responsive, reduction-responsive, temperature-responsive, and photoactive micelles.

Among various stimuli-sensitive block copolymers, the pH-sensitive micelles mainly consist of acrylic acid (AA) [87,88], 2-diisopropylaminoethyl methylacrylate (DPA) [89], and 2-(dimethylamino) ethyl methacrylate (DMAEMA) [90,91] blocks. 

#### 2.1.1. pH-Sensitive Micelles

**Figure 1:**
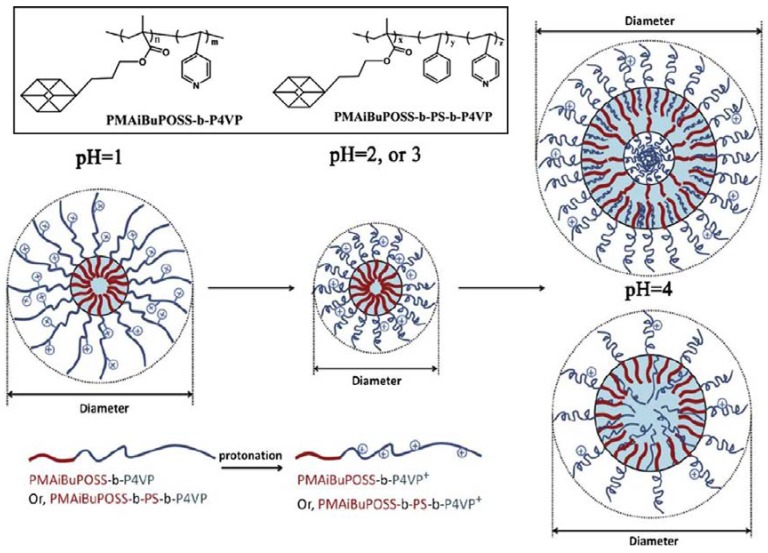
Schematic of the variation in pH-sensitive micelles of HBCP. Reprinted with permission from [92]. Copyright (2013) Elsevier.

A series of POSS-containing pH-sensitive block copolymers (HBCP), comprised of poly(methacrylisobutyl-POSS)-*b*-poly(4-vinylpyridine) (PMAiBuPOSS-*b*-P4VP) and poly(methacrylisobutyl-POSS)-*b*-polystyrene-*b*-poly(4-vinylpyridine) (PMAiBuPOSS-*b*-PS-*b*-P4VP), were synthesized via reversible addition fragmentation chain transfer (RAFT) polymerization by Xu and coworkers [92]. The self-assembly behavior of the HBCP is attributed to a balance of three kinds of driving forces: the hydrophobic interaction of POSS, the electrostatic interaction of P4VP blocks, and the π–π stacking interaction of the pyridine groups. Low pH values enable the P4VP block to become highly protonated, resulting in a larger hydrodynamic size of the aggregates due to the strong electrostatic repulsion-induced molecular stretch (Figure 1). Increase of the pH value resulted in more curled molecular chains of P4VP because of a decrease in the electrostatic repulsion of the P4VP. When the pH value further increases close to the pK_a_ of P4VP, the protonation degree of the P4VP block is lowered enough to change P4VP to a hydrophobic state. Consequently, a stronger π–π stacking interaction leads to the formation of larger HBCP aggregates.

Another example reported by the same group is poly(methacrylate isobutyl POSS)-*b*-poly(3-dimethyl(methacryloyloxyethyl) ammonium propane sulfonaten-*co*-2-(diethylamino) ethyl methacrylate-*co*-styrene) (PMAiBuPOSS-*b*-P(DMAPS-*co*-DMAEMA-*co*-St) prepared by the ring-opening reaction [69]. The copolymer can be assembled into relatively uniform micelles in water. The size of the micelles is determined by the length of the DMAEMA block, pH value, and the content of DMAPS; the longer DMAEMA blocks yield a larger size than that of shorter DMAEMA blocks because of the better hydrophilicity of the chains. Besides, the micelle size also exhibited a great dependence on pH, which decreased with the increasing of the pH value. Furthermore, higher content of DMAPS showed strong electrostatic interactions, which weaken the interactions of the molecular chains. Thus, the micelles become more compact due to the molecular chains’ shrinkage.

**Figure 2:**
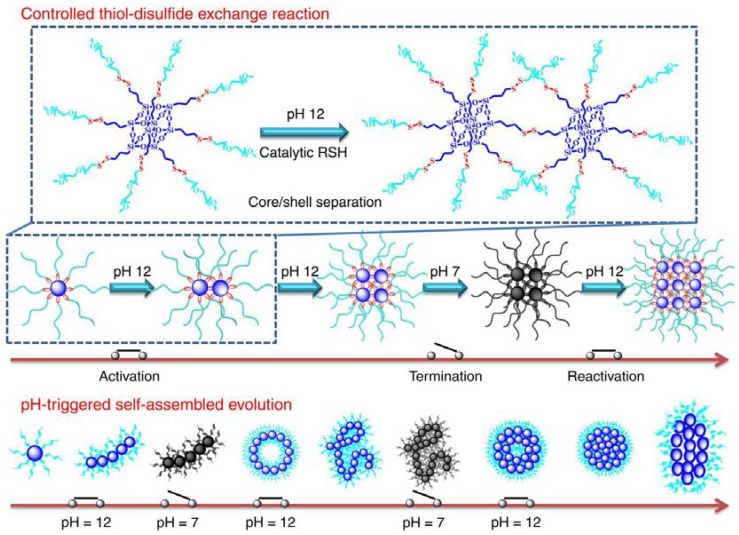
Controllable morphology evolution of the copolymers with a pH-switched on/off function. When the thiol–disulfide exchange reaction is activated in the presence of catalytic thiols in pH 12 solutions, the selective core/shell separation gradually leads to the continuous connections of POSS-embedded cores. Then, the self-assembled evolution is performed from unimolecular micelles to form the elliptic nanoparticles. Reprinted with permission from [93]. Copyright (2018) Creative Commons Attribution 4.0 International License.

A special pH- and reduction-sensitive micelle was reported by Wang and coworkers [93]. By activation and/or termination exchange reaction of the POSS–(SSPEG)_8_ copolymer via the thiol–disulfide bonds, they could modulate the constructive linkage of POSS-embedded segments into micelles and return back through PEG segments (Figure 2). The activated POSS–(SSPEG)_8_ acted as the preassemblies for post micellar connection, axial growth, bending, and cyclization processes driven by the highly active connection points. Then, the preassemblies could provide a platform for the formation of all the nanoscale, microscale, and macroscale morphologies. In this system, the rigid POSS-embedded backbone with strong aggregation tendency can promote the hybrid polymers with unique self-assembly behaviors, and thereby various hierarchical nanostructures can be further generated.

#### 2.1.2. Thermosensitive Micelles

In general, amphiphilic block copolymers can self-assemble in selective solvents to form micelles. However, thermoresponsive polymers are completely soluble in the solvent in all proportions at temperatures below the lower critical solution temperature (LCST) and become insoluble above the LCST. The thermosensitive micelles mainly consist of *N*-isopropylacryl amide (NIPAM) [90,94,95,96], propylene glycol [67,97], ε-caprolactone [98,99,100], and oligo(ethylene glycol) methacrylate (OEGMA) [101,102,103].

**Figure 3:**
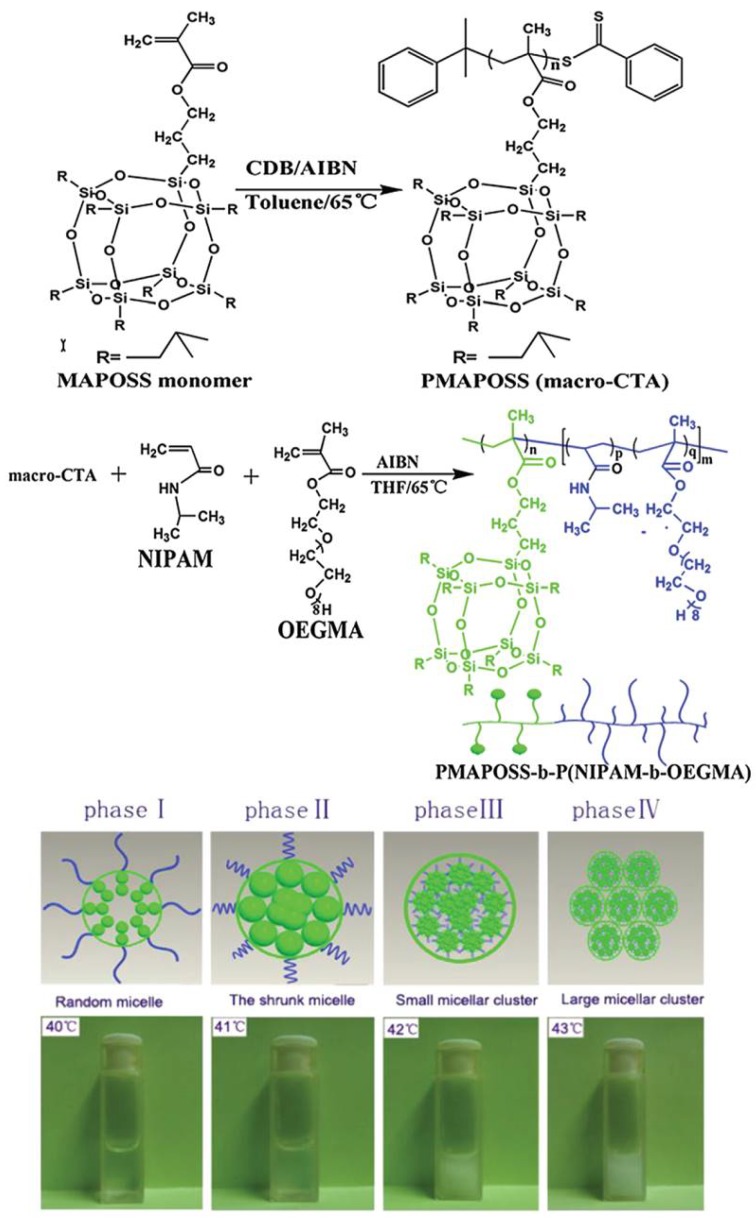
Synthetic route of the PMAPOSS-*b*-P(NIPAM-*co*-OEGMA) block copolymer via reversible addition fragmentation chain transfer (RAFT) polymerization and images of the phase changes for the PMAPOSS-*b*-P(NIPAM-*co*-OEGMA) micelle with solution heating and the macroscopic phase transition of PMAPOSS_9_-*b*-P(NIPAM_180_-*co*-OEGMA_15_) solution with the temperature ranging from 40 °C to 43 °C. CDB, AIBN, MAPOSS and macro-CTA represents for cumyl dithiobenzoate, 2,2′-azoisobutyronitrile, methacrylate isobutyl POSS and PMAPOSS, respectively. Reprinted with permission from [104]. Copyright (2014) John Wiley and Sons.

Poly(methacrylate isobutyl POSS)-*b*-poly(*N*-isopropylacrylamideco-oligo(ethylene glycol) methyl ether methacrylate) (PMAPOSS-*b*-P(NIPAM-*co*-OEGMA) was synthesized via RAFT polymerization (Figure 3). Due to the hydrophobic nature of the PMAPOSS segment and hydrophilic nature of the p(NIPAM-*co*-OEGMA) segment, it can self-assemble into spherical micelles. The essentially predetermined sharp and intensive LCST can be modulated by adjusting the content of NIPAM or OEGMA domains. In addition, these hybrid micelles could be reversibly associated or disassociated by heating and cooling the solution with several cycles, and the degree of reversibility is greatly concentration-dependent (Figure 3) [104]. 

**Figure 4:**
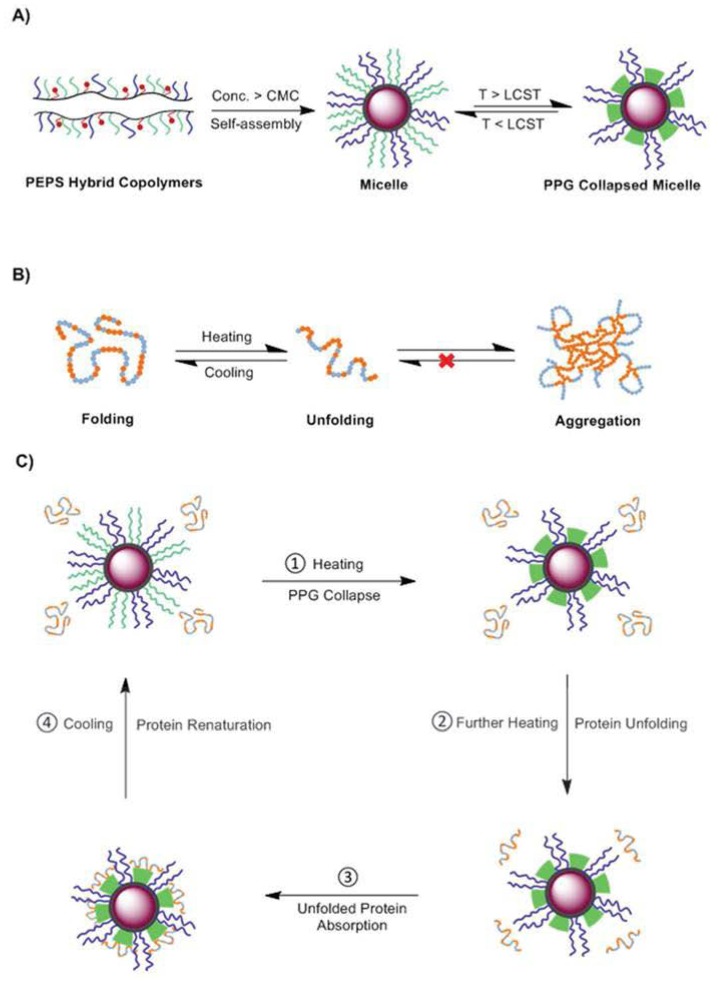
Schematic representation of (**A**) thermoresponsive poly(PEGMA-PPGMA-POSSMA) (PEPS) hybrid copolymer self-assembly in aqueous solution and (**B**) heat-induced protein denaturation process. Yellow and light blue spots represent the hydrophobic and hydrophilic sites of proteins, respectively. (**C**) Proposed working mechanism of the thermally denatured protein protection by thermoresponsive PEPS hybrid micelles. LCST, CMC and PPG represents for lower critical solution temperature, critical micelle concentration and poly(propylene glycol), respectively. Reprinted with permission from [67]. Copyright (2014) The Royal Society of Chemistry.

Poly(propylene glycol) (PPG) is another thermally responsive polymer with tunable hydrophilic–hydrophobic properties triggered by external temperature. The phase transition can be tuned with temperature ranging from 14 °C to 100 °C, depending on the architecture and molecular weight, which makes it more attractive in temperature-responsive self-assemblies. Hybrid copolymers prepared from poly (ethylene glycol) methacrylate (PEGMA) and methacrylate POSS (POSSMA) together with poly (propylene glycol) methacrylate (PPGMA) were reported by Li and coworkers [67]. The synthesized poly (PEGMA–PPGMA–POSSMA) (PEPS) exhibited LCST ranging from 31 to 33 °C. Static and dynamic light scattering (SLS and DLS) studies showed core-shell micellar morphologies (Figure 4). Compared to samples without POSS, PEPS copolymers with only 3.1 wt % POSS could effectively lower the critical micelle concentration (CMC) of the micelles at room temperature by one order of magnitude. In addition, PEPS with 6.7 wt % POSS exhibited constant hydrodynamic radius (R_h_ = 65 nm) and aggregation number (N_agg_ = 350) when temperature was varied from 20 to 70 °C. They found that such interesting findings of PEPS hybrid copolymers could open up new opportunities to protecting unfolded proteins from aggregation under high temperatures.

Star-shaped polymeric micelles with poly(ε-caprolactone)-poly(2-(2-methoxyethoxy)ethyl methacrylate)-*co*-poly(ethylene glycol) methacrylate) (POSS-(PCLP( MEO_2_MA-*co*-PEGMA))_16_) were synthesized via ATRP, ROP, and click reactions [98]. Owing to the hydrophobic property of POSS and poly(ε-caprolactone) (PCL) cores and the hydrophilic p(MEO_2_MA-*co*-PEGMA) segments, the amphiphiles were found to self-assemble into ellipsoidal structures with a moderately uniform size. In addition, the thermoresponsive properties could be finely tuned by changing the feed ratios of MEO_2_MA and PEGMA. 

**Figure 5:**
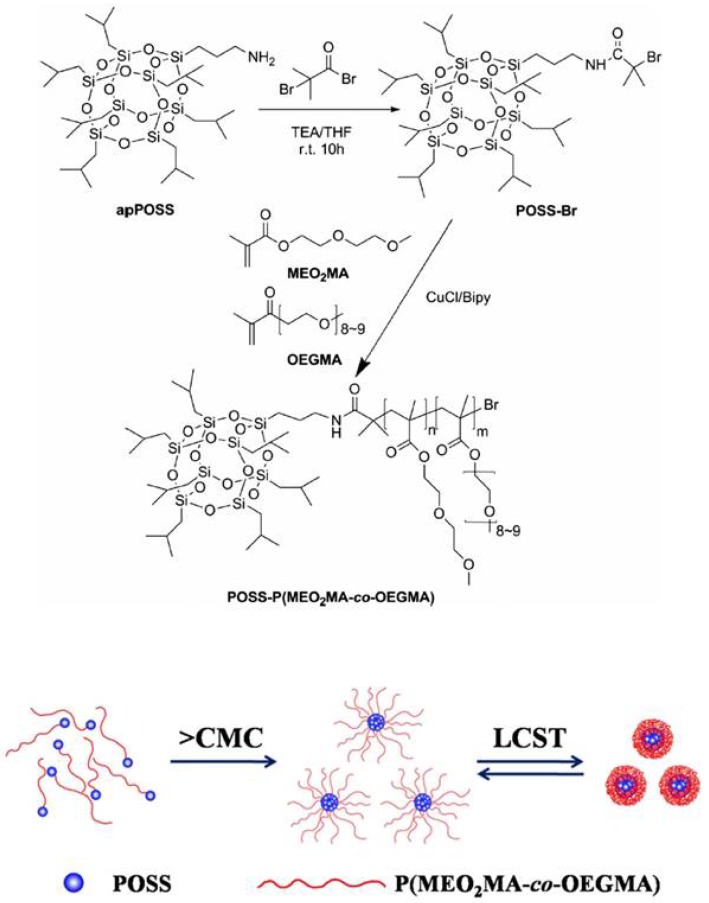
Synthesis of thermoresponsive POSS–P(MEO_2_MA-*co*-OEGMA) and schematic self-assembly process of the POSS–P(MEO_2_MA-*co*-OEGMA) in water in response to temperature. Reprinted with permission from [105]. Copyright (2014) Springer Nature.

Li and coworkers found that the solution behaviors of POSS–P(MEO_2_MA-*co*-OEGMA) are mainly determined by the balance between hydrophilic and hydrophobic moieties. At low temperature, the amphiphilic P(MEO_2_MA-*co*-OEGMA) formed hydrogen bonds with water, whereas the POSS showed a competitive hydrophobic effect. However, this balance was disrupted when temperature was higher than the LCST. The dehydration interaction induced the formation of large aggregates which, in turn, would self-assemble into a core–shell nanostructure with the hydrophobic POSS as the core of the micelles and the hydrophilic P(MEO_2_MA-*co*-OEGMA) as the corona of the micelles (Figure 5) [105]. 

#### 2.1.3. Photoactive Micelles

**Figure 6:**
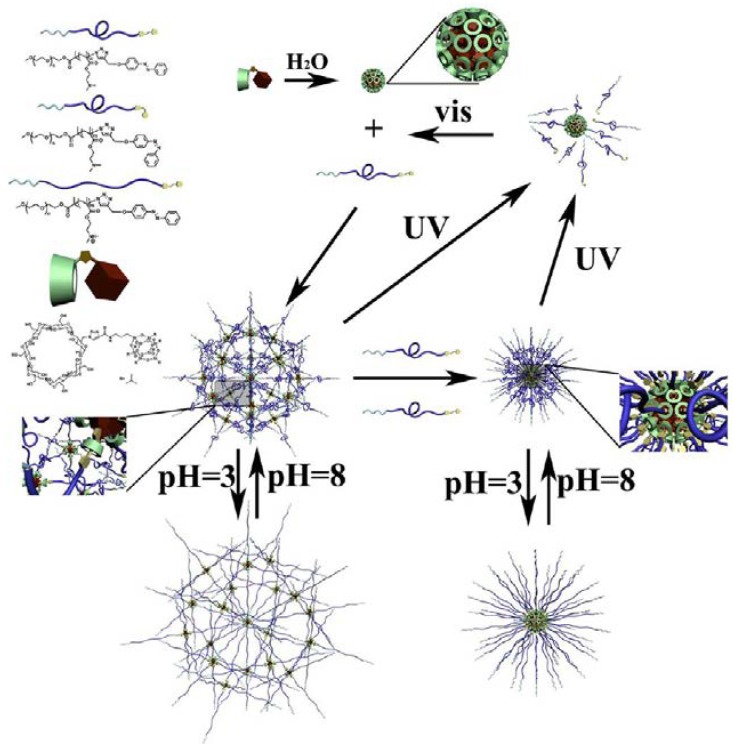
Proposed mechanism of the self-assembly process between PEG-*b*-PDMAEMA-azo (PPA) and mono-cyclodextrin substituted isobutyl polyhedral oligomeric silsesquioxane (mCPOSS) in aqueous solution. Reprinted with permission from [106]. Copyright (2014) American Chemical Society.

The optically sensitive micelles are normally triggered by photoresponsive chromospheres. A smart multi-stimuli-responsive copolymer (PEG-*b*-PDMAEMA-azo, PPA) of monocyclodextrin-substituted isobutyl POSS (mCPOSS) and azobenzene end-capped poly(ethylene glycol)-*b*-poly (2-(dimethylamino) ethyl methacrylate) was prepared (Figure 6) [106]. The mechanism of its micelle formation in aqueous solution started from the nanosphere formation of mCPOSS, self-assembled because of its amphipathic property. Then, the trans-azo end groups on PPA interacted with the cyclodextrin, followed by the supramolecular assembly between PPA and mCPOSS. The morphology, formation/dissociation, and size could be adjusted by the ratio of PPA and mCPOSS, visible and ultraviolet light, and pH, respectively.

### 2.2. Other Mechanisms in Different Assemblies 

#### 2.2.1. Micelles

**Figure 7:**
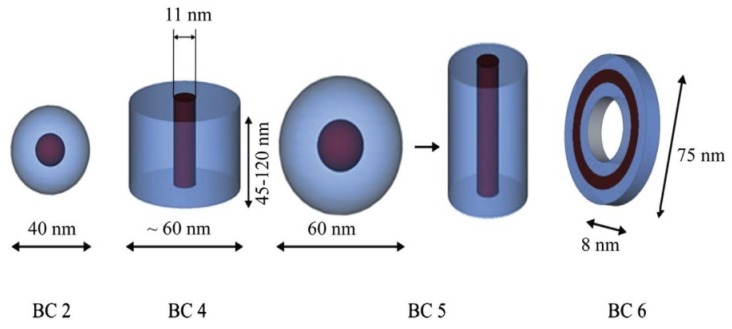
The increase in the POSS block fraction in the block copolymer (BC) BC2 (1 mol% POSS) < BC5 (4 mol% POSS) < BC4 (9 mol% POSS) < BC6 (11.65 mol% POSS) series leads to different morphologies. Reprinted with permission from [109]. Copyright (2014) Elsevier.

Aminopropylisobutyl POSS (ap-POSS–Br)-initiated polymerization of methylmethacrylate (MMA) and methacrylisobutyl POSS (MAPOSS) was reported to produce self-assembly into 150–300-nm core–shell micelles with ap-POSS/MA-POSS as the core and poly methylmethacrylate (PMMA) as the shell, or core–shell–crown micelles with poly methacrylisobutyl POSS (P(MA-POSS)) as the core, PMMA as the shell, and ap-POSS as the crown when P(MA-POSS) content was increased [107]. It was found that POSS is a powerful hydrophobic unit in the assembly of poly (acrylic acid)-*co*-poly(acrylate-POSS) [108]. With low POSS ratios in the copolymers, i.e., 40 to 110 acrylic acid repeat units to one POSS, the copolymers could self-assemble to form nanoaggregates. Increasing the volume fraction of insoluble blocks could result in morphological transformations (Figure 7) [109]. The solvent effect mentioned showed that the copolymer POSS-*b*-PDMAEMA-*b*-PMMA self-assembled into polymeric micelles with different shapes such as spherical, rod, and necklace morphologies in various solutions [110]. In specific solvents, P(MMA-*co*-GMA)-*b*-PMAPOSS could form ordered micelle-like structures such as spherical, cylindrical, or vesicle-like morphologies by tuning the copolymer and mixed solvent compositions [109]. Controlling the length of the particular blocks also makes it possible to tune the micellar structures. Copolymers with short-range ordering generally lead to spherical micelles or cylindrical and lamellar aggregates. 

**Figure 8:**
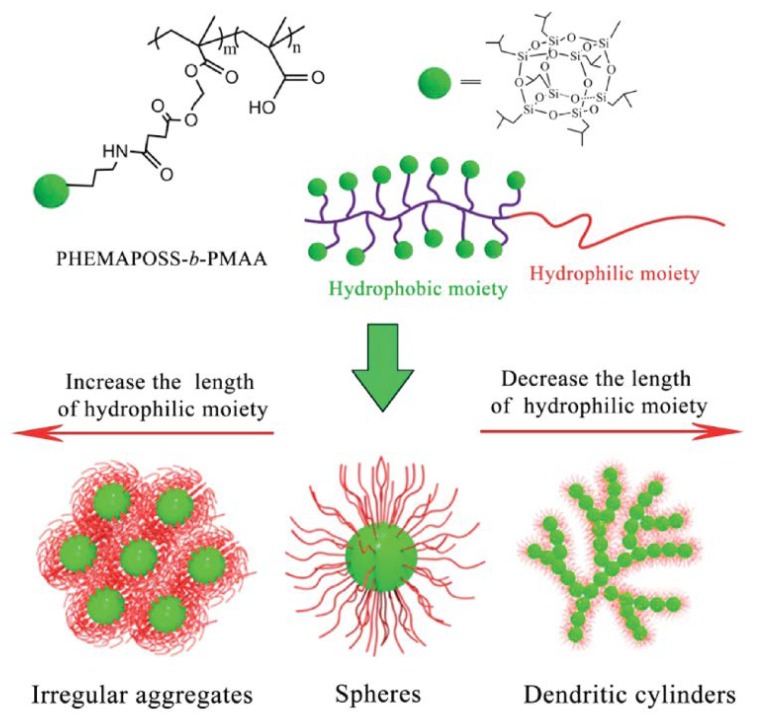
Self-assembly behaviors of PHEMAPOSS-*b*-PMAA in water with different lengths of hydrophilic PMAA moieties. Reprinted with permission from [35]. Copyright (2014) American Chemical Society.

Zhang and coworkers studied the chain length effect of poly(2-hydroxyethyl methacrylate-POSS)-*b*-(poly(methyl methacrylate))(PHEMAPOSS-*b*-PMAA) possessing different lengths of hydrophilic chains [35]. The PHEMAPOSS_45_-*b*-PMAA_523_ formed typical core–shell spherical micelles with the hydrophobic poly(2-hydroxyethyl methacrylate-POSS) (PHEMAPOSS) blocks as the core and hydrophilic PMAA blocks as the shell. The micelles are not conventional core–shell micelles because of the dispersion of POSS moieties in the aggregates. Longer PMAA chains of PHEMAPOSS_45_-*b*-PMAA_1173_ resulted in irregular aggregates after self-assembly, whereas shorter hydrophilic PMAA chains of PHEMAPOSS_45_-*b*-PMAA_308_ led to dendritic cylinder assemblies. Thus, the assembled morphologies of PHEMAPOSS-*b*-PMAA block copolymers can be mediated (Figure 8). 

In addition, electrostatic interaction between the two kinds of micelles was reported in the fabrication of mixed micelles as well [111].

#### 2.2.2. Spheres

**Figure 9:**
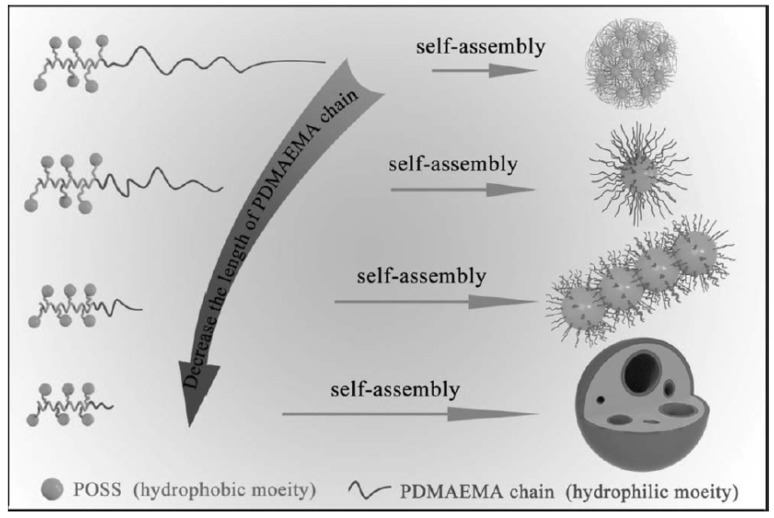
Self-assembly of poly(2-hydroxyethyl methacrylate-POSS)-*b*-(poly(methyl methacrylate)) (PHEMAPOSS-*b*-PDMAEMA) in water with the decreasing length of the hydrophilic PDMAEMA chain. Reprinted with permission from [114]. Copyright (2014) John Wiley and Sons.

A novel poly(ε-caprolactone)-block-poly(butadiene-g-POSS)-block-poly(ε-caprolactone) (denoted as PCL-*b*-P(B-*g*-POSS)-*b*-PCL) amphiphilic block copolymer was found to self-assemble into spherical microdomains with sizes of 20−50 nm in the epoxy matrix [112]. The amount of the microdomains increased proportionally with the increasing content of PCL-*b*-P(B-*g*-POSS)-*b*-PCL. Due to the different viscoelastic properties of the poly(B-*g*-POSS) segments of the organic polymers, the P(B-*g*-POSS) blocks separated from the epoxy network to form dispersed microdomains, whereas PCL blocks remained miscible in the epoxy matrix during the processing of epoxy resins. Meng and coworkers prepared multiple cluster-wrapped polymers and block copolymers via the ROP reaction [113]. The incorporation of POSS clusters as side groups was found to affect the size of assemblies due to the stretching effect of the polymer chains. To study the chain length effect, Zhang and coworkers prepared various PHEMAPOSS-*b*-PDMAEMA copolymers with different lengths of PDMAEMA blocks via RAFT reaction [114]. By varying the length of hydrophilic PDMAEMA blocks, the morphologies of the self-assembled structures can be tuned from irregular aggregates to spherical core–shell micelles and further from pearl-necklace-like structures to capsules (Figure 9).

**Figure 10:**
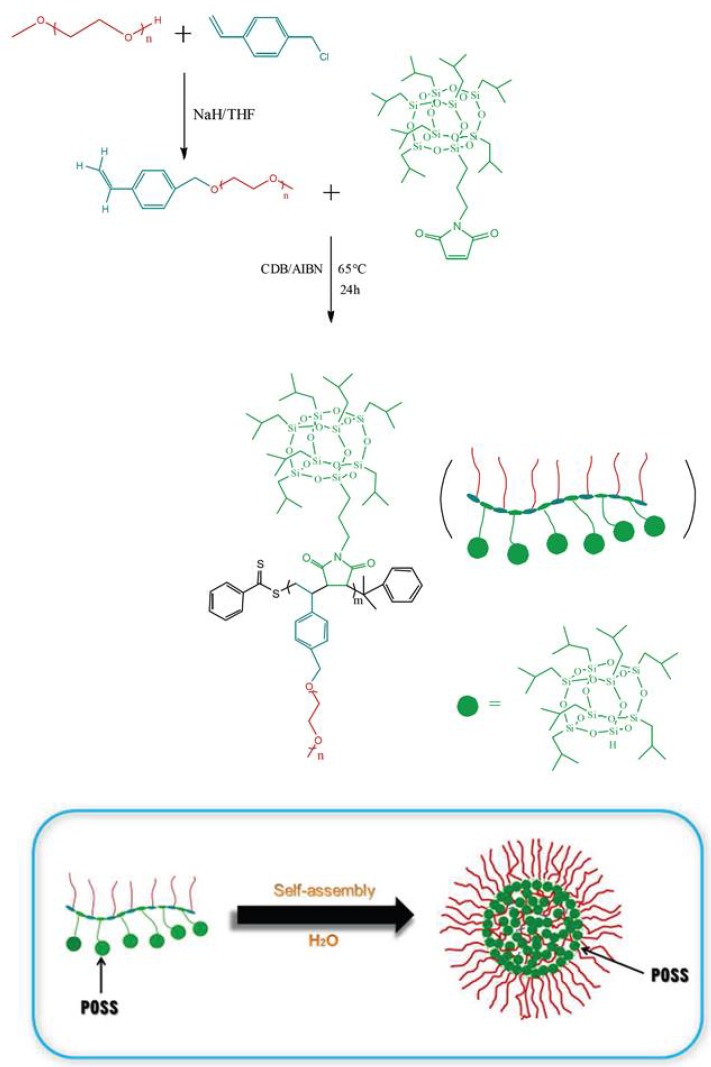
Schematic illustration of the synthesis of poly(maleimide isobutyl polyhedral oligomeric silsesquioxane-*alt*-vinylbenzyl polyethylene glycol) (P(MIPOSS-*alt*-VBPEG)) by RAFT polymerization and self-assembly of P(MIPOSS-*alt*-VBPEG)_2_. Reprinted with permission from [37]. Copyright (2015) the Centre National de la Recherche Scientifique (CNRS) and The Royal Society of Chemistry.

Later, they reported the synthesis of hybrid alternating copolymer brushes using maleimide isobutyl POSS (MIPOSS) and 4-vinylbenzyl-terminated polyethylene glycol (VBPEG) via RAFT polymerization (Figure 10) [37]. Results showed that all the alternating copolymers can form spherical aggregates with size ranging from 70 to 200 nm. Moreover, the average size of the spherical aggregates decreases with the increase of the chain length of the hydrophilic PEG segment. Instead of forming typical micelles with hydrophobic POSS moieties as the core and hydrophilic PEG chains as the shell, the core of these spherical aggregates may contain some PEG chains, except for POSS moieties, which resulted from the alternating structure of poly(maleimide isobutyl polyhedral oligomeric silsesquioxane-*alt*-vinylbenzyl polyethylene glycol) (P(MIPOSS-*alt*-VBPEG)) copolymers.

#### 2.2.3. Sheets

Generally, block copolymers are able to form a variety of ordered nanostructures via self-assembly. Specially, POSS-*b*-PEO (poly(ethylene oxide)s) block copolymers were reported by Yu and coworkers to crystallize in a selected solvent, which drives the block copolymers to organize into large nanothick sheets [115]. The sheet size can be modulated ranging from micrometers to tens of micrometers by increasing the ratio of the POSS in the samples. The sheet formation was attributed to a balance between the PEO block crystallization and the solubility of the POSS block in the mixed solvent. The solubility of the whole POSS-*b*-PEO block polymer with 5 K molecular weight of PEO (nPOSS-*b*-5.0kPEO) in the mixed solvent was found to increase with the increasing content of POSS, along with the slowing down of sheet growth and the decrease of the dimensions.

**Figure 11:**
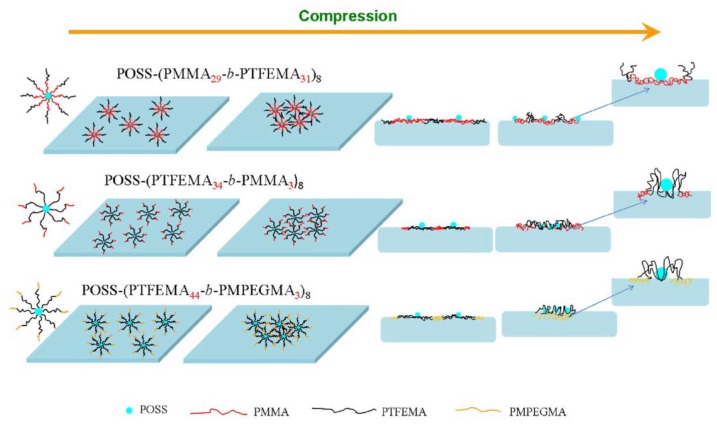
Proposed packing model of the three-copolymer self-assembly at the air–water interface. Reprinted with permission from [116]. Copyright (2016) Springer Nature.

Li and coworkers proposed and verified the packing model of POSS–(PMMA-*b*-PTFEMA)_8_, POSS–(PTFEMA-*b*-PMMA)_8_, and POSS–(PTFEMA-*b*-PMPEGMA)_8_) at the air–water interface by the Langmuir–Blodgett method [116]. With compression, all the molecules occupied a large surface area at the beginning and the POSS adopted a condensed globular state. The copolymer chains exhibited an almost 2D conformation and the elasticity increased with the surface concentration. Further increasing surface pressures beyond the elasticity maximum, the hydrophilic blocks (PMPEGMA) or hydrophobic blocks (PMMA) would protrude into the water subphase and the elasticity values would decrease (Figure 11). Further compression led to stronger interaction of the copolymer, and the molecules were densely packed to a brush conformation. 

## 3. Applications

### 3.1. Drug Delivery

**Figure 12:**
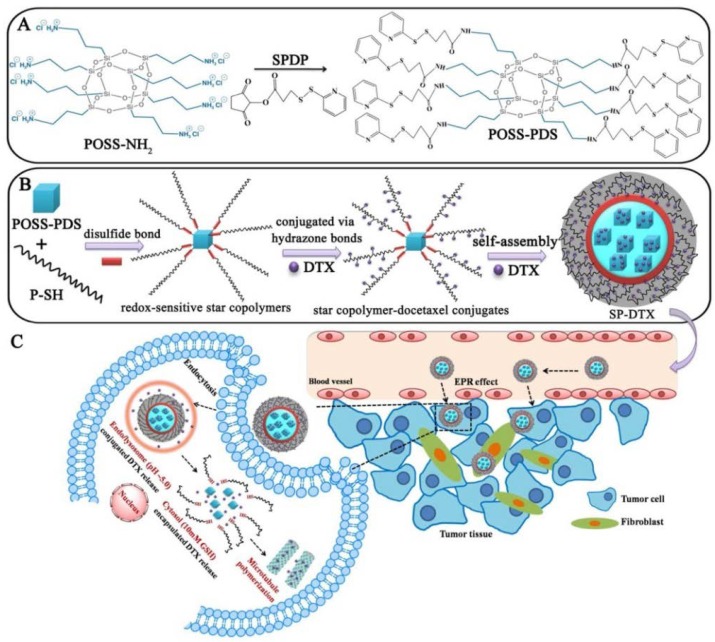
Schematic illustration of (**A**) the synthesis procedure of pyridyldisulfanyl-functionalized POSS (POSS-PDS). (**B**) Self-assembly of SP-DTX nanoparticles from amphiphilic star-shaped POSS-based conjugates. (**C**) Tumor accumulation and intracellular trafficking pathway of SP-DTX nanoparticles. SPDP represents for *N*-succinimidyl 3-(2-pyridyldithio) propionate. Reprinted with permission from [124]. Copyright (2016) American Chemical Society.

Drug delivery with self-assembled amphiphilic copolymers has been extensively studied in the past two decades due to the ease of synthesis and structure modulation [4,117,118]. Among various drugs, drugs for cancer treatment and diagnosis have attracted increasing attention [119,120]. As a drug carrier, stability is quite important because once the micelle disassembles into free polymer chains, it will result in a burst release of encapsulated drugs, which is not so desirable for clinic usage [121,122]. To endow carriers with controlled delivery, Li and coworkers have reviewed the development of various hybrid nanocarriers in remotely triggered drug release [123]. In terms of POSSs, Yang and coworkers reported a star-like organic–inorganic conjugate of a POSS-based nanomedicine [124]. The hybrids were synthesized by grafting semitelechelic *N*-(2-hydroxypropyl) methacrylamide (HPMA) copolymers to POSS through reductively degradable disulfide bonds (Figure 12A). The anticancer drug docetaxel (DTX) was attached to the grafts via pH-sensitive hydrazone bonds and also encapsulated into the POSS. Such star-shaped conjugates could self-assemble into nanoparticles SP-DTX (the grafts attached with hydrophobic docetaxel (DTX) by pH-sensitive hydrazone bonds and encapsulated into the POSS core) and exhibited conspicuous drug-loading capacity (20.1 wt %) (Figure 12B). The stimuli-responsive DTX release under acidic lysosomal and reducing cytoplasmic environments was verified as well. SP-DTX also displayed uniform tumor distribution and tumor growth inhibition of 78.9%, compared to that of non-redox-sensitive SP-DTX-A (67.4%), in contrast to the SP-DTX-C, which contained DTX only in the core and exhibited only 65.5% inhibition, and linear P-DTX, which showed 60.7% suppression through enhanced depletion of cancer-associated fibroblasts and induction of apoptosis (Figure 12C). The star-shaped POSSs show advantages in increased drug-loading capacity, uniform particle distribution, and enhanced drug stability.

Other pH-sensitive micelles were prepared with poly(ε-caprolactone)-poly(2-(dimethylamino)ethyl methacrylate)-*co*-poly(ethylene glycol) methacrylate) (POSS-PCL-P(DMAEMA-*co*-PEGMA))_16_, using PCL as cores and star-shaped P(DMAEMA-*co*-PEGMA) as coronas and a dialysis process [125]. Different concentrations of micelles could be obtained during dilution. The triggered self-assembly behavior of these triblock copolymers could be modulated by pH values from 5.0 to 7.4 for controlled doxorubicin release. The drug release efficiency reached up to 82% (*w*/*w*) with identification of the location of the doxorubicin (DOX) in HeLa cells and no associated cytotoxicity.

**Figure 13:**
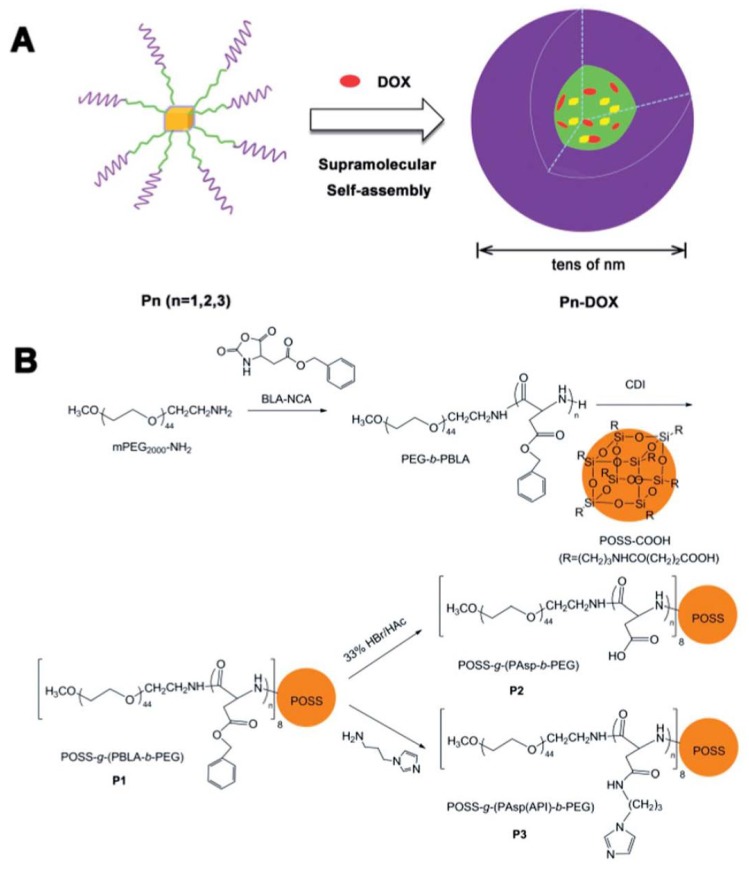
Schematic illustration of the DOX-loaded micelle formation (**A**) and the synthetic route of the star-shaped copolymers (**B**). P_n_ represents for poly(benzyl l-aspartate)-*b*-poly(ethylene glycol) (PBLA-*b*-PEG) copolymers. Reprinted with permission from [28]. Copyright (2014) The Royal Society of Chemistry.

Amphiphilic poly(l-aspartate)-*b*-poly(ethylene glycol) block copolymers were synthesized and immobilized onto POSS and to obtain star-shaped POSS-*g*-(PBLA-*b*-PEG) copolymers [28]. 1-(3-aminopropyl) imidazole was grafted to the pendant groups of poly (l-aspartate) to fabricate pH-sensitive micelles (Figure 13). Due to hydrophobic and π–π interactions, these star-shaped copolymers could self-assemble into micelles with size ranging from 100–200 nm in an aqueous medium, and DOX was trapped inside the micelle. The release study shows (Figure 13) that the release of DOX-loaded micelles with imidazole groups was pH-dependent, and more than 90% of the loaded DOX could be released within 48 hours in a weakly acidic medium (pH 5.0). 

A similar report was obtained in the case of star-shaped amphiphilic block copolymers (poly (benzyl-l-aspartate)-block-poly(ethylene glycol)) with POSSs as the cores, which could self-assemble into micelles in an aqueous medium as well [126]. The drug loading content and encapsulation efficiency increases with increasing chain length of the PBLA blocks when quercetin is used as a model.

### 3.2. Photodynamic Therapy

**Figure 14:**
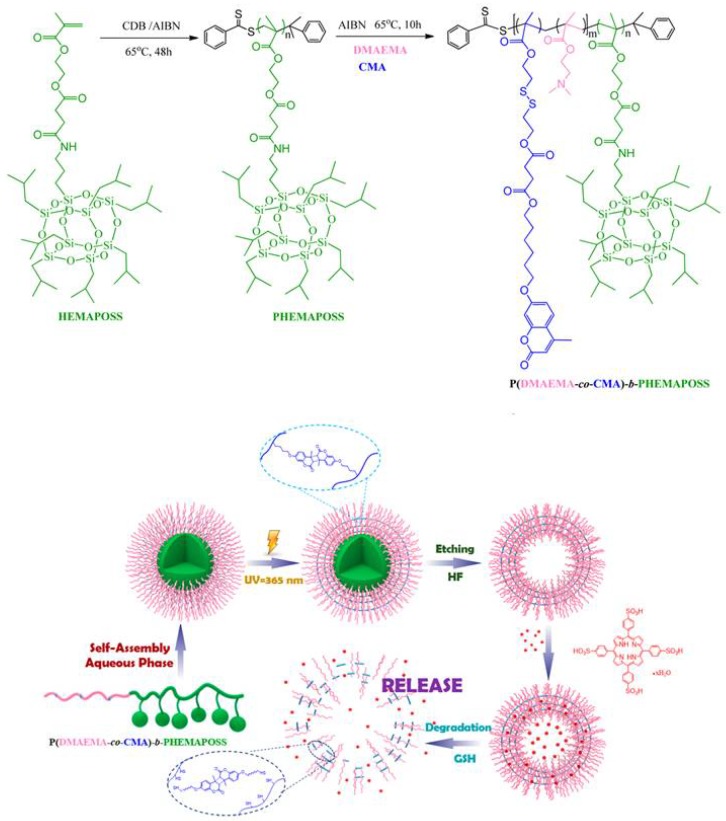
Schematic illustration of the synthesis of the PHEMAPOSS-*b*-P(DMAEMA-*co*-CMA) block copolymer and structural changes of polymeric capsules in the process of loading and release of TPPS. Reprinted with permission from [127]. Copyright (2016) American Chemical Society.

A novel amphiphilic diblock copolymer, poly(methacrylate monomer based on polyhedral oligomeric silsesquioxane) block poly(dimethylamino ethyl methacrylate-*co*coumarin methacrylate) PHEMAPOSS-*b*-P(DMAEMA-*co*-CMA), was prepared for photodynamic therapy (PDT) application via RAFT polymerization (Figure 14) [127]. Photodimerization of coumarin led to the formation of micelles with POSS cores and stimuli-responsive shells, and then hollow polymeric capsules could be finally obtained via etching the POSS core (Figure 14). The hollow polymeric capsules are multi-stimuli-responsive to redox potential and pH and could be utilized in the encapsulation and release of tetraphenylporphyrin tetrasulfonic acid hydrate (TPPS). The capsule showed a relatively low TPPS release at pH = 7.4. However, a burst release of TPPS was found in the presence of 10 mM glutathione (GSH) at pH = 5.5. TPPS-loaded polymeric capsules also demonstrated low dark toxicity toward MCF-7 cells. Due to the size effect of POSS, it could be an ideal sacrificial template for the encapsulation of drug molecules.

**Figure 15:**
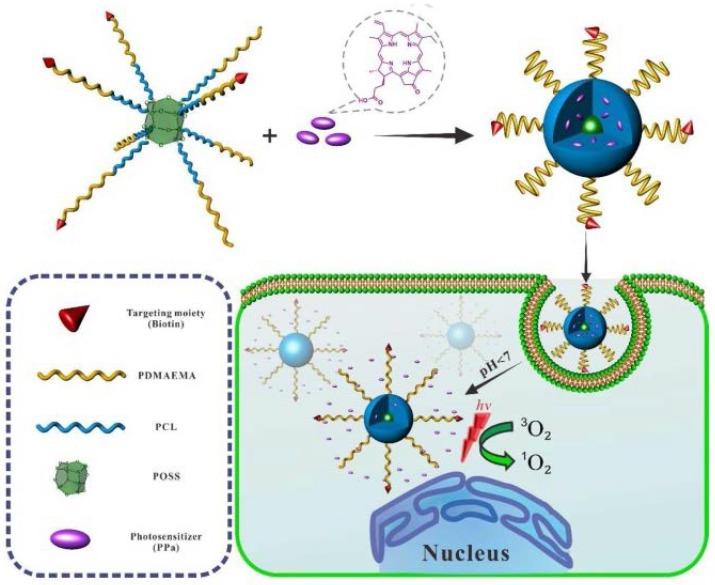
Construction of unimolecular micelles and the process of photodynamic therapy (PDT). Reprinted with permission from [128]. Copyright (2017) Elsevier.

Later, the same group reported stable unimolecular micelles self-assembled from POSS–(PCL-*b*-PDMAEMA)_8_–biotin with an inorganic POSS nucleus, a hydrophobic poly(ε-caprolactone) (PCL) middle layer, and a hydrophilic poly(2-(dimethylamino)ethyl methacrylate) (PDMAEMA) outer corona (Figure 15) [128]. The micelles were utilized in the encapsulation and release of hydrophobic pheophorbide A (PPa) photosensitizers for photodynamic therapy (PDT). PPa-loaded tumor-targeted micelles could boost the internalization rate in HeLa cells effectively. In addition, the micelles also showed low dark toxicity and high PDT efficacy towards HeLa cells according to the 3-(4,5-dimethylthiazol-2-yl)-2,5-diphenyltetrazolium bromide (MTT) assay.

### 3.3. Coating

Due to the intrinsic properties of POSS molecules, the motion of the chains can be effectively controlled and therefore induce molecules to self-assemble, which could provide the copolymer with promising properties when used as coatings. Furthermore, POSS groups were reported to show low surface energy and thus could migrate and aggregate on the coating surfaces, endowing them with hydrophobicity for antifouling/anti-icing [129,130,131]. 

**Figure 16:**
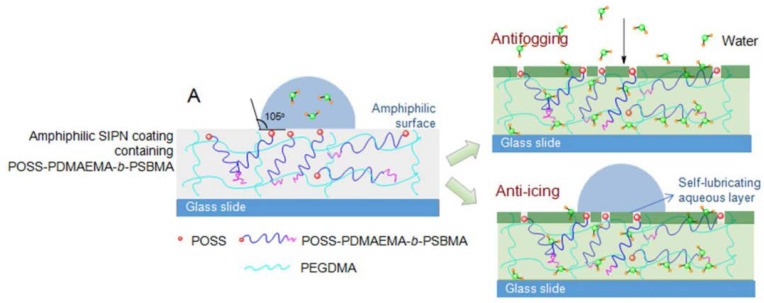
Schematic illustration of the semi-interpenetrating polymer network (SIPN) coatings containing POSS–PDMAEMA-*b*-PSBMA with POSS aggregated on the surface and the strongly hydrophilic chains distributed beneath them. Reprinted with permission from [132]. Copyright (2017) American Chemical Society.

Li and coworkers developed highly transparent antifogging/anti-icing coatings with POSS–poly(2-(dimethylamino)ethyl methacrylate)-block-poly(sulfobetaine methacrylate) (POSS-PDMAEMA-b-PSBMA) with a small amount of ethylene glycol dimethacrylate (EGDMA) (Figure 16) [132]. The POSS clusters aggregated and dispersed well within the polymer matrix with the size of 10–80 nm. With the hygroscopicity of both PDMAEMA and PSBMA blocks with polymerization of EGDMA and the hydrophobicity of POSS, the copolymers exhibited excellent antifogging properties. In addition, the hygroscopic coatings could manipulate water molecules well-dispersed into the hydrophilic matrix via hydrogen-bonding interactions. Interestingly, the amphiphilic coatings exhibited the anti-icing ability with a freezing delay time of more than 2 min at −15 °C, owing to the aggregation tendency of hydrophobic POSS and the self-lubricating aqueous layer generated by the nonfreezable water on the surface. 

### 3.4. LB Films

**Figure 17:**
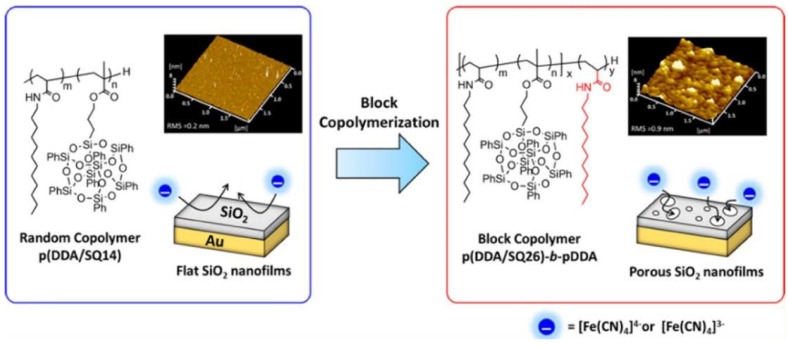
Schematic illustration of the preparation of the hybrid block copolymer consisting of *N*-dodecyl acrylamide (DDA) and silsesquioxane (SQ) comonomers (p(DDA/SQ26)-*b*-pDDA) and the assembled Langmuir–Blodgett (LB) nanofilms as well as the porous nanofilms. Reprinted with permission from [77]. Copyright (2018) American Chemical Society.

As an effective method in creating three-dimensional nanostructures by assembling a two-dimensional molecular layer at the air–water interface, the Langmuir–Blodgett (LB) technique is considered to be the most widely utilized one [133,134]. In the past few years, POSS-based materials have been recognized as promising materials forming stable Langmuir films [135,136,137,138]. For example, Ishizaki and coworkers prepared a porous SiO_2_ nanofilm by using hybrid block copolymers consisting of N-dodecyl acrylamide (DDA) and silsesquioxane (SQ) comonomers (p(DDA/SQ26)-*b*-pDDA) (Figure 17) [77]. Photo-oxidation of the amphiphiles of the Langmuir−Blodgett (LB) film leads to the porous film formation. These p(DDA/SQ26)-*b*-pDDA monolayers exhibited high collapse pressure (π_c_ = 45.8 mN·m^−1^) and good monolayer stability. So, this method of photo-oxidation of the self-assembled hybrid block copolymers is a promising method for manipulating pore formations of inorganic oxide nanofilms.

### 3.5. Optical Sensors

**Figure 18:**
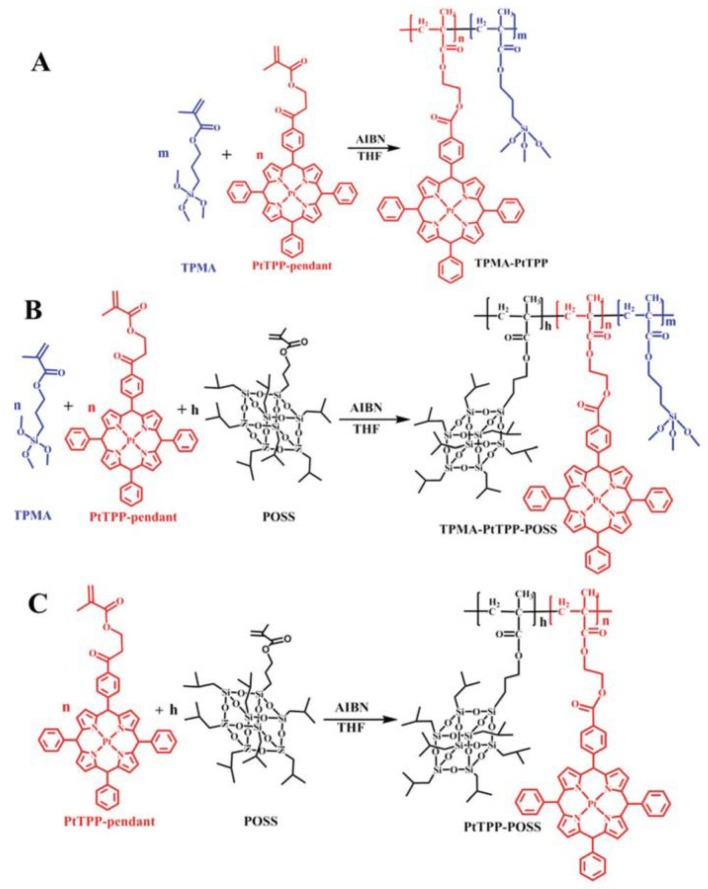
Syntheses of the oxygen-sensitive copolymers of TPMA–PtTPP (**A**), TPMA–PtTPP–POSS (**B**), and PtTPP–POSS (**C**) copolymers. Reprinted with permission from [139]. Copyright (2017) The Royal Society of Chemistry.

Organic–inorganic hybrid 3-(trimethoxysily)propylmethacrylate-*co*-platinum porphyrin-*co*-methacrylolsobutyl-polyhedral oligomeric silsesquioxane (TPMA–PtTPP–POSS) copolymer films were prepared and applied as high-performance oxygen sensors (Figure 18) [139]. The strong repulsive interactions between the organic (TPMA) and inorganic POSS blocks of the copolymers allow the formation of ordered nanostructures with smaller feature sizes. The introduction of POSS could enhance surface roughness and surface area for sensing. The oxygen sensor showed high sensitivity, with a quencher rate coefficient (K_SV_) of 1.833 kPa^−1^, and swift response capability (0.6 s). Such high sensitivity was ascribed to the wormlike structures of the copolymers. The structure ensured the homogeneous dispersion of PtTPP, which could be perturbed by a trace oxygen environment. More similar works could be developed in the area of the sensing of other gases, gas-responsive coatings, or gas-sensitive hydrogels, or perhaps even self-healing materials.

As reported by Chen and coworkers, a metal-sensitive organic–inorganic hybrid amphiphile consisting of a POSS hydrophobic head and a hydrophilic PEG tail functionalized along with a bidendate ligand was synthesized [140]. Results indicated that the hybrid was thermally instable, and the thermal instability of the hybrids might have resulted from a decrease in the hydrogen bonding between PEG chains as well as between PEG and water. However, the metal–ligand coordination was strong enough to stabilize the micelles in the Zn^2+^/POSS-MA-PEG-DPA system, although the hydrogen bond interactions were damaged. Accordingly, their results suggest that the micelle morphology of POSS-MA-PEG-DPA in solution was sensitive to Zn^2+^ due to the coordination reaction. Thus, this new type of amphiphile indicates potential application in the fabrication of metal-sensitive sensors.

## 4. Conclusions and Perspectives

In this review, we have demonstrated that POSSs are one of the most interesting and important building blocks to construct and drive the assembly of amphiphilic hybrid POSS copolymers. The key features of POSSs in building the amphiphilic copolymers and their assembly come from its unique hybrid cage structures with uniform size in the -molecular level (1–3 nm core), together with its interesting physical properties such as hydrophobic and stable Si–O cores with functional peripheral organic groups. As we have illustrated in the abovementioned examples, interactions for self-assembly of the amphiphilic copolymers also include simple amphiphilic assembly and it can be increasingly effective when specific Van der Waals forces are introduced. However, one of the challenges in this area which still remains to be resolved is the incorporation of bioactive segments onto these copolymers, which would allow more specificity and targeting for triggered release. In addition, it would be more meaningful if the assemblies can be prepared uniformly in batches for preclinical and clinical tests for developing practical applications. Moreover, great opportunities still remain for the development of diversiform self-assembly systems, because various functional siloxane precursors are now commercially available and also precise control of the substitution is possible due to the synthetic flexibility.
